# Clinical and Laboratory Features of Enteric Fever in Children and Antibiotic Sensitivity Pattern in a Tertiary Care Hospital of a Low- and Middle-Income Country

**DOI:** 10.7759/cureus.30784

**Published:** 2022-10-27

**Authors:** Nadia Nusrat, Md. Rafiqul Islam, Nibedita Paul, Neshwa Rahman, Ambigga Krishnapillai, Md. Ahsanul Haq, Mainul Haque

**Affiliations:** 1 Pediatrics, Delta Medical College & Hospital, Dhaka, BGD; 2 Primary Care Medicine, Faculty of Medicine and Defence Health, National Defence University of Malaysia, Kuala Lumpur, MYS; 3 Biostatistics, International Centre for Diarrhoeal Disease Research, Bangladesh (ICDDR, B), Dhaka, BGD; 4 Pharmacology and Therapeutics, National Defence University of Malaysia, Kuala Lumpur, MYS

**Keywords:** blood culture, bangladesh, developing countries, teaching hospital, form, antimicrobial sensitivity, paediatric, typhoid fever, laboratory aspects, symptoms and signs

## Abstract

Background: Globally, enteric fever (EF) significantly gives rise to an appalling death toll. It is an endemic illness in Bangladesh and South Asia. The condition manifests in a wide range of clinical features in children. Nowadays, antibiotic resistance is an international stumbling block that hampers the appropriate treatment and outcome of EF.

Objective: The study evaluated the clinical and laboratory characteristics and antibiotic sensitivity pattern of *Salmonella enterica* in children.

Methods: This prospective research was conducted at Delta Medical College and Hospital, Dhaka, Bangladesh, from January 2017 to December 2019. A total of 200 pediatric cases of EF were included in this study who were either culture positive or had significantly raised Widal test titer for *Salmonella *with suggestive clinical features.

Results: All the patients had a fever, and most had coated tongue, vomiting, abdominal pain, organomegaly, and diarrhea. Among the selected 200 cases of EF, 43.5% were *Salmonella typhi* culture-positive. A high erythrocyte sedimentation rate (ESR) was observed in a substantial number (53%) of patients. Ceftriaxone was the most sensitive (100%) antibiotic through laboratory analysis, followed by cefotaxime (95.1%). Among the oral antibiotics used, cefixime (92.8%) was the most sensitive.

Conclusion: EF in children can present with varied clinical manifestations. Selective antibiotic treatment according to sensitivity patterns is crucial for effective illness management and will reduce morbidity and mortality in the pediatric population.

## Introduction

Typhoid fever (TF) continues to be a substantial public health problem in developing countries [1]. The disease is caused by *Salmonella typhi* and *paratyphi*. Both are members of the Enterobacteriaceae family [2]. *S. typhi* grows only in humans and is transferred through the fecal-oral route. Every year, it is estimated internationally, that 215,000 death results from over 26 million cases of EF and five million cases of paratyphoid infection [3]. The incidence of EF is more common in low- and middle-income countries (LMICs) [4], especially in Bangladesh, the Indian subcontinent, South and Central America, and Southern Africa, than in developed countries [1,5-8]. The EF is characterized by fever, which frequently causes headaches, dry cough, and myalgia [9,10]. Abdominal features in most patients manifest as abdominal pain, constipation, or diarrhea [11]. Relative bradycardia develops when the EF disease process continues for seven days or more with a febrile ailment [3]. Additionally, the spleen is often palpable with abscess formation [12,13]. Other studies reported hepatic [14] and ovarian [15] abscess formation. Rose spots appear in around one-quarter of patients [16,17]. Children under five years commonly report diarrhea, nausea, febrile seizures, and prominent neurological manifestations [18-20]. EF results in delirium [21], obtundation [22], intestinal hemorrhage [23], and bowel perforation when the disease process prolongs to one month and remains untreated [24].

Blood culture has remained the standard method [25] for the diagnosis of EF since 1907 [26,27]. Nevertheless, at most, only 45%-70% of corroborating cases are identified [28], and this diagnosis process is slow and consumes quite a few days [25]. Moreover, the sensitivity of blood culture possesses several issues, including a declining trend as typhoid disease progress [29, 30]. Stool and rectal swab cultures play a substantial role in diagnosing EF [31] and have the possibility to provide affirmative results by the third week of EF [3]; regrettably, serological tests for EF, inclusive of the Widal test, are all impeded by high rates of false-positive along with false-negative results [32-35].

Internationally, multidrug-resistant (MDR) strains appeared in the second half of the 1980s. Chloramphenicol, ampicillin, and co-trimoxazole were regarded as the first-line therapy of EF [36]. The imprudent utilization of antimicrobials is prevalent around the globe, explicitly in LMICs, and has promoted the selective pressure and propagation of antimicrobial-resistant strains [37]. Furthermore, newer antimicrobials were poorly developed, accountable to oppressive regulatory requirements and reduced financial encouragement [38]. Moreover, it has been reported that in Bangladeshi hospitals that 80% of antimicrobials were prescribed imprudently. In addition, 70% were antimicrobials among the total prescribed medications [39]. Furthermore, it has been reported that in Bangladesh, the chicken industry widely and irrationally prescribed antibiotics to enhance poultry growth and egg production [40]. One more Bangladeshi study revealed that 64.28% of isolates of *S. typhi* were multidrug resistant [8].

Objectives of the study

This study was conducted to evaluate the clinical and laboratory profile of EF in children and to learn about the antibiotic sensitivity pattern of *Salmonella*, which will help properly choose antibiotics and thus reduce morbidity and mortality in the pediatric population.

## Materials and methods

Study type, place, and period

This is a prospective study, which was conducted at the Department of Pediatrics, Delta Medical College & Hospital, Dhaka, Bangladesh. This study was conducted for three years from January 2017 to December 2019.

Study population 

Inclusion Criteria

A total of 200 children aged one to 15 years who were either blood culture positive for the Fastidious Antibiotic Neutralization (FAN) or had significant Widal test titer (at least four-fold rises or 1:160 dilution of both O and H antibodies) were included in the study.

Blood Culture

After the blood collection, it was inoculated in blood agar or MacConkey agar medium and observed for the organism's growth. If the organism's growth occurs, the colony is transferred to Mueller-Hinton agar, a microbiological growth medium commonly used for antibiotic susceptibility testing, specifically disk diffusion test. Then, an antibiotic was given at a specific distance, and a sensitivity report was given depending on the bacterial inhibition zone [41].

Widal Test

After centrifugation of blood, serum was collected and transferred to a tube. Then, Widal test reagents (containing *Salmonella typhi* and *paratyphi* antigen) were added and observed for agglutination. Reports were given depending on antibody titer. A titer of 1:160 or more was considered significant [42].

Exclusion Criteria

Those who had enteric fever with comorbidities (malignancy, nephrotic syndrome, chronic kidney disease, chronic liver disease, etc.) or complications (multiorgan failure, encephalopathy, etc.) were excluded from the study.

Data collection

The research participants' clinical history was recorded as per standard regulation. The required clinical and laboratory information was collected in the preformed spreadsheet.

Data analysis

Data were processed and evaluated using computer software called Statistical Package for Social Sciences (SPSS; IBM Corp., Armonk, NY).

Ethical approval

This research obtained ethical approval from the Institutional Review Board (IRB) of Delta Medical College & Hospital, Dhaka, Bangladesh, with the reference number DLMCH/IEC/2020/1 on October 15, 2020. The study subjects were initially verbally informed about the study design, purpose, future publication, and their right to withdraw from the project at any time for any reason. Subjects who had given written informed consent to participate in the study were included.

## Results

A total of 200 children with EF were comprised in this research. Among them, 117 (58.5%) were male, and 83 (41.5%) were female. Most EF cases (48%) were in the age group of less than five years. Table 1 shows the age-wise distribution of patients. The average age was 4.13 years, and the lowest age was one year. Fever was present in all patients (100%). Other important findings were coated tongue (35.5%), hepatomegaly (34%), vomiting (25.5%), abdominal pain (21.5%), diarrhea (16.5%), and splenomegaly (16%). Table 2 shows the clinical features of the patients with sex distribution.

**Table 1:** Distribution of cases (n = 200) according to age and sex

Age	Male	Female	Total
1-5 years	56 (28%)	40 (20%)	96 (48%)
>5-10 years	45 (22.5%)	32 (16%)	77 (38.5%)
>10-15 years	16 (8%)	11 (5.5%)	27 (13.5%)

**Table 2:** Principal clinical characteristics of enteric fever with sex distribution Notes: An enlarged spleen was detected through a physical examination (abdominal palpation).

	Overall	Male (117)	Female (n = 83)	p-value
Clinical features				
Fever	200	117 (100%)	83 (100%)	
Coated tongue	71 (35.5%)	43 (36.8%)	28 (33.7%)	0.660
Hepatomegaly	67 (33.5%)	37 (31.6%)	30 (36.1%)	0.505
Vomiting	51 (25.5%)	33 (28.2%)	18 (21.7%)	0.297
Abdominal pain	43 (21.5%)	26 (22.2%)	17 (20.5%)	0.768
Diarrhea	33 (16.5%)	17 (14.5%)	16 (19.3%)	0.373
Splenomegaly	32 (16.0%)	18 (15.4%)	14 (16.9%)	0.778

**Figure 1:**
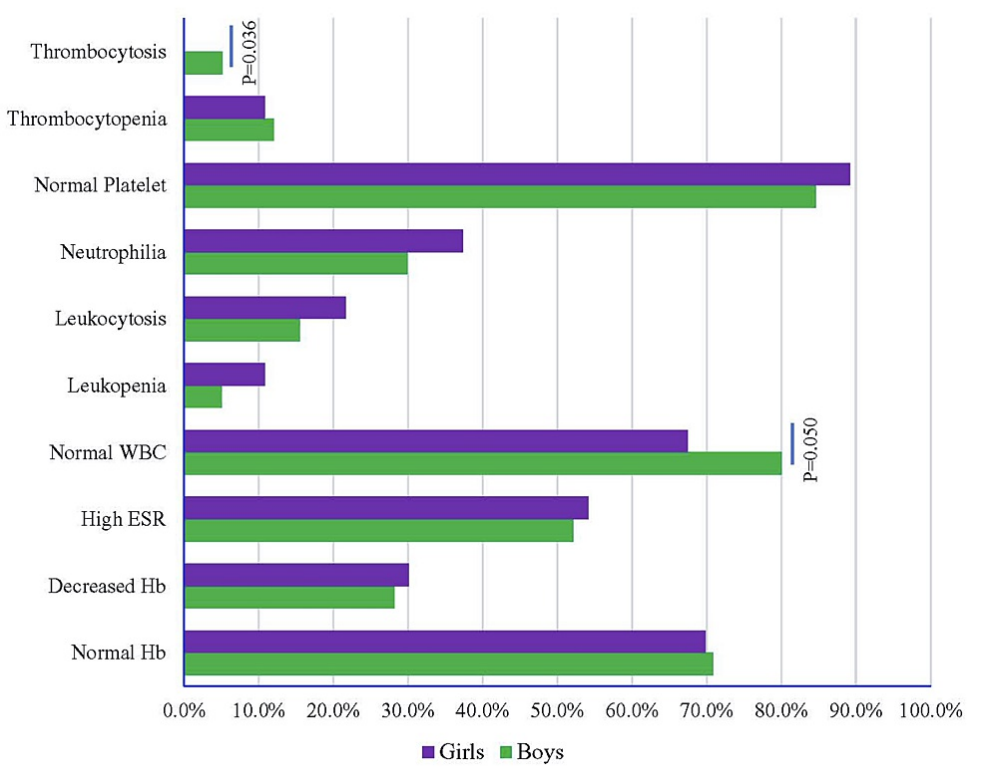
Laboratory findings of enteric fever with sex distribution ESR: Erythrocyte sedimentation rate.

**Figure 2:**
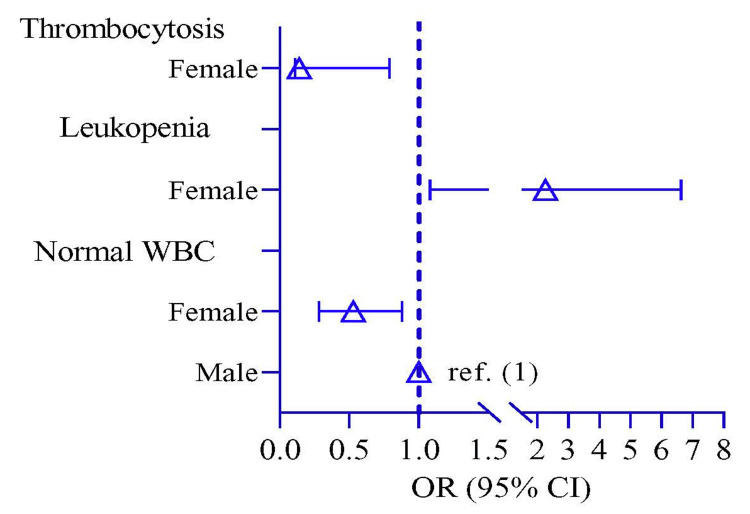
Risk of enteric fever among female children compared to males

Figure 1 shows the laboratory findings of EF with sex distribution. Normal WBC was significantly higher in 93 male patients (80.0%) compared to females (p = 0.050). Odds of normal WBC and thrombocytosis were lower in the female participants by 0.53 times (p = 0.044, 95% CI: 0.28, 0.88) and 0.14 times (p = 0.035, 95% CI: 0.11, 0.79), respectively, compared to male participants. Thrombocytosis showed a higher risk in male participants by 2.27 times (p = 2.27, 95% CI: 1.08, 6.63) compared to female participants (Figure 2, Table 3).

**Table 3:** Odds of laboratory findings of enteric fever in females compared to males Notes: The logistic regression model was used to estimate the odds ratio (OR) and p-value. The regression model was adjusted by age. In the regression model, the OR represented the OR risk of female participants compared to male participants.

Key Findings	OR (95% CI)	p-value
Normal WBC		
Boys	Ref.	
Girls	0.53 (0.28, 0.88)	0.044
Leukopenia		
Boys	Ref.	
Girls	2.27 (1.08, 6.63)	0.047
Thrombocytosis		
Boys	Ref.	
Girls	0.14 (0.11, 0.79)	0.035

Culture positivity was 43.5%, and 56.5% of patients had significantly raised Widal test titer. Among the culture-positive 87 cases, 84 (96.55%) were *S. typhi*, and three (3.44%) were *S. paratyphi*. Table 4 shows the antibiotic sensitivity pattern of *Salmonella*. Ceftriaxone was found to be the most sensitive antibiotic (100%), followed by cefotaxime (95.1%) and ceftazidime (91.5%). Cefixime was the most sensitive among oral antibiotics (92.8%). Nalidixic acid was our study's least sensitive (18.30%) drug. Ciprofloxacin and ceftazidime had significantly higher resistance (26.2% and 14.6%, respectively) in males compared to females (Table 4).

**Table 4:** Antibiotic sensitivity pattern in overall participants Notes: Data were presented as numbers with percentages in the parenthesis. Chi-square test was used to estimate the p-values. Additionally, chi-square is only applicable when we have dichotomous or 2 x 2 contingency tables.

Antibiotics	Overall	Male (117)	Female (n = 83)	p-value
Ampicillin				
Sensitive	47 (56.6%)	22 (52.4%)	25 (61.0%)	0.722
Resistance	34 (41.0%)	19 (45.2%)	15 (36/6%)	
Intermediate resistance	2 (2.41%)	1 (2.38%)	1 (2.44%)	
Amoxycillin				
Sensitive	47 (56.6%)	24 (57.1%)	23 (56.1%)	0.923
Resistance	36 (43.4%)	18 (42.9%)	18 (43.9%)	
Intermediate resistance				
Chloramphenicol				
Sensitive	67 (80.7%)	32 (76.2%)	35 (85.4%)	0.289
Resistance	16 (19.3%)	10 (23.8%)	6 (14.6%)	
Intermediate resistance				
Cotrimoxazole				
Sensitive	63 (75.9%)	30 (71.4%)	33 (80.5%)	0.335
Resistance	20 (24.1%)	12 (28.6%)	8 (19.5%)	
Intermediate resistance				
Ciprofloxacin				
Sensitive	65 (78.3%)	31 (73.1%)	34 (82.9%)	0.004
Resistance	17 (21.7%)	11 (26.2%)	7 (17.0%)	
Intermediate resistance				
Cefixime				
Sensitive	77 (92.8%)	38 (90.5%)	39 (95.1%)	0.607
Resistance	4 (4.82%)	3 (7.14%)	1 (2.44%)	
Intermediate resistance	2 (2.41%)	1 (2.38%)	1 (2.44%)	
Ceftriaxone				
Sensitive	83 (100.0%)	42 (100%)	41 (100%)	
Resistance				
Intermediate resistance				
Ceftazidime				
Sensitive	75 (91.5%)	37 (90.2%)	40 (97.6%)	0.048
Resistance	5 (6.10%)	4 (9.80%)	1 (2.44%)	
Intermediate resistance				
Cefotaxime				
Sensitive	78 (95.1%)	38 (92.7%)	40 (97.6%)	0.305
Resistance	4 (4.90%)	3 (7.30%)	1 (2.40%)	
Intermediate resistance				
Nalidixic acid				
Sensitive	15 (18.3%)	7 (17.1%)	8 (19.5%)	0.775
Resistance	67 (81.7%)	34 (82.9%)	33 (80.5%)	
Intermediate resistance				
Gentamicin				
Sensitive	68 (82.9%)	35 (85.4%)	33 (80.5%)	0.795
Resistance	10 (12.2%)	4 (9.76%)	6 (14.6%)	
Intermediate resistance	4 (4.88%)	2 (4.88%)	2 (4.88%)	
Azithromycin				
Sensitive	43 (53.1%)	21 (52.5%)	22 (53.7%)	0.619
Resistance	19 (23.5%)	8 (20.0%)	11 (26.8%)	
Intermediate resistance	19 (23.5%)	11 (27.5%)	8 (19.5%)	

**Figure 3:**
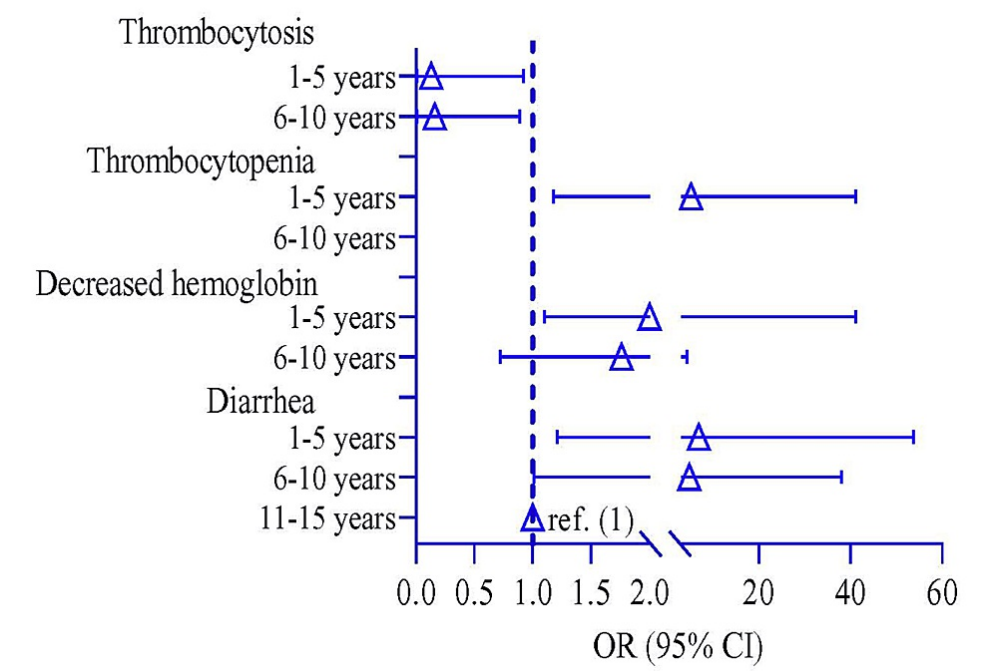
Risk of enteric fever among young children compared to adolescents

Participants were divided into three groups based on their age: 1-5 years, 6-10 years, and 11-15 years. A logistic regression model observed the risk of clinical symptoms among the different age groups, where 11-15 years was a reference group. The risk of diarrhea was higher in 6-10 years by 4.80 times (p = 0.049, 95% CI: 1.01, 38.0) and 1-5 years by 6.85 times (p = 0.041, 95% CI: 1.21, 53.7) compared to 11-15 years of age children (Table 5). The risk of decreasing hemoglobin and thrombocytopenia was higher in children 1-5 years of age by 2.0 times (p = 0.046, 95% CI: 1.10, 4.93) and 5.21 times (p = 0.038, 95% CI: 1.18, 41.2), respectively, compared to that of the 11-15 years. In contrast, the risk of thrombocytosis was lower in children 1-5 years old by 0.13 times (p = 0.038, 95% CI: 0.01, 0.92) compared to 11-15 years (Table 5 and Figure 3).

**Table 5:** Symptomatic risk of enteric fever among young children compared to adolescents The logistic regression model was used to estimate the odds ratio (OR) and p-value. The regression model was adjusted by age. In the regression model, the OR represents the OR risk of children in the 1-5 years and 6-10 years groups compared to 11-15 years group.

Symptoms	OR (95% CI)	p-value
Diarrhea		
11-15 years	Ref.	
6-10 years	4.80 (1.01, 38.0)	0.049
1-5 years	6.85 (1.21, 53.7)	0.041
Decreased hemoglobin		
11-15 years	Ref.	
6-10 years	1.76 (0.72, 4.27)	0.212
1-5 years	2.00 (1.10, 4.93)	0.046
Thrombocytopenia		
11-15 years	Ref.	
6-10 years	2.20 (0.26, 19.2)	0.475
1-5 years	5.21 (1.18, 41.2)	0.038
Thrombocytosis		
11-15 years	Ref.	
6-10 years	0.16 (0.01, 0.89)	0.031
1-5 years	0.13 (0.01, 0.92)	0.038

## Discussion

In this prospective study, boys were affected more than girls, with a male-female ratio of 1.4:1, compatible with other studies [1,43-49]. Rabasa AI et al. found male-female ratio of 3:2 in their study [50]. In multiple studies, EF was found considerably higher in females than males [51,52]. Nevertheless, another study's sex ratio was almost equal [53]. Our study's mean age was 4.13 years, slightly lower than that of another study [45]. The lowest age was only one year in the current study. In our study, most patients were less than five years, which is consistent with another study [44]. Other studies found that most patients were aged 5-10 years [1,45-47,49-51,54-56]. Other studies reported that EF patients were common at 10 years or older [53,57]. Fever was the usual clinical presentation seen in all patients, which was consistent with earlier multiple research [43-46]. The next common feature was coated tongue (35.5%). This finding of coated tongue in multiple studies was slightly higher [43,46,49,52,56]. Nonetheless, the coated tongue was much higher (81.63%) in another study [45] but was less common (14.9%) in one more research [54].

In our study, vomiting and abdominal pain were present in 25.5% and 21.1% of patients, respectively. In other studies, vomiting was found in a similar percentage of patients [45,54]. The number was much higher (71.4%) in the study conducted by Rabasa Al et al. [50], whereas it was found in a minimal number of patients (9%) in the study conducted by Islam et al. [43]. Our study's findings on abdominal pain were similar to other studies [44,46,49]. Still, the number is much higher in other studies [43,45,47,52,54]. We found hepatomegaly in 34% of patients. The current study finding was similar to earlier studies [52,54,56]. Laishram et al. [45] and Singh et al. [47] found hepatomegaly in many patients, which is 77.5% and 79.7%, respectively. Splenomegaly was found in 16% of patients in our study. The same finding was observed in other studies [46,47], but the number was higher in other studies [45,54]. Sudarshan [49] found splenomegaly in 68.4% of patients, which is much higher. Diarrhea was present in 16.5% of patients, which was consistent with other studies [43,46,47,49], but it was slightly higher in other studies [44,45,54]. Punjabi et al. found diarrhea commonly in paratyphoid fever [57].

Most (70.5%) of this study population had a normal hemoglobin level, consistent with earlier studies [46,52,53]. Reduced hemoglobin was found in 29.5% of patients, but it is much higher in the studies done by Sudarshan [49], Sarswat et al. [56], and Behera et al. [58]. We found high ESR in 53% of patients, which was higher in another study [59]. The WBC count was normal in most patients (74.5%), which is consistent with other studies [47,49,58]. Leukopenia was found in 7.5% of patients, which was similar to studies conducted by other authors [52,53,56,58], but it was much higher (34%) in the study done by Devaranavadagi and Srinivasa [46]. Leukocytosis was seen in 18% of patients, consistent with other studies [46,47,52,56,58]. Neutrophilia was found in our study in 33% of cases, which is consistent with other studies [46]. Lymphocytosis was present in 20% of patients. This finding was much more in other studies [52,53]. Behera et al. [58] found eosinopenia in their study's large number of patients (58.93%). This finding was also present in other studies' small number of patients [46,56]. The majority of patients (86.5%) showed normal platelet count. Thrombocytopenia was found in 11.5% of our patients. A similar finding was found in other studies [46,58], but in some studies, this finding is much higher [53,56]. Al Reesi et al. reported a case of a four-year-old boy with severe thrombocytopenia (platelet 16 × 10^9^/L) with EF [60].

The current study revealed that blood culture was positive for *Salmonella* in 43.5% of cases, similar to other studies [47-49,54]. Saha et al. [44] found culture positivity in higher number of patients (62%). The finding is much lower in other studies [52,61] among culture-positive cases. *S. typhi* was present in 96.55% of cases, and *S.* *paratyphi *was present in 3.44% of cases. This is consistent with the study by Kamaal et al. [54]. The percentage of *paratyphi *is slightly higher in other studies [1,44,48,57,61,62]. Kuijpers et al. found *paratyphi *more than *typhi *in their study [63]. Paratyphoid fever was more common in children below two years in another study conducted by Punjabi et al. [57].

Ceftriaxone showed 100% sensitivity in all isolates, similar to other studies [46-49,64]. But few cases were resistant to this drug in other studies [52,54]. In our research, cefotaxime was the second most sensitive drug (95.1%). Sensitivity to this drug is slightly lower in other studies [46,54]. Cefixime showed 92.8% sensitivity. This antibiotic showed 100% sensitivity in other studies [46,48,56,64] but was least sensitive in another study [47]. A significant tendency of drug resistance was designated for nalidixic acid and fluoroquinolones from 2006 to 2015 [65]. Nalidixic acid showed only 18.30% sensitivity in our study. The drug is sensitive in 50% of cases in the study done by Singh et al. [47] but shows resistance in 80% of subjects in one review [66] and is resistant in 100% of patients in another study [58,64]. *S. paratyphi* was sensitive to ceftriaxone, cefixime, cotrimoxazole, chloramphenicol, and ciprofloxacin in all cases, consistent with another study [54]. *S. paratyphi* showed greater antimicrobial resistance than *typhi *in the study conducted by Punjabi et al. [57], and Sudarshan [49] found 53.6% of cases of MDR EF in their research. Still, in another study, it is 15% [64]. Judio et al. [55] reported that all cases were sensitive to first-line drugs, and no MDR case existed. MDR and quinolone-resistant strains of *S. typhi *and *paratyphi A* were predominant among travelers coming back from Asia [67]. *S. typhi *and *paratyphi *strains' resistance types diverge in different areas of South Asia. However, a decline in MDR strains of EF has been demonstrated in India and Bangladesh; nevertheless, fluoroquinolone resistance patterns were steadily increasing. In contrast, there is a preponderance of MDR strains in Pakistan and Nepal and evolving extensively drug-resistant (XDR) strains in Pakistan [68].

Limitations of the study

The limitation of the study was the diagnosis of EF using the Widal test or clinical parameters having low specificity. There was the possibility of many cases being falsely labeled as typhoid. Patients with EF may have negative results in blood culture, so some cases were missed. This is another limitation. Bone marrow culture was not done in any patients. Socio-economic status and vaccination history were not taken in this study.

## Conclusions

EF is an endemic disease in most developing countries, particularly in South-East Asia. Drug resistance has already emerged as a public health threat in developing and developed countries. More studies should be done to identify the resistant organism and sensitivity pattern of antimicrobials in different areas to select appropriate medication to treat EF. Additionally, worldwide antimicrobial stewardship programs should be introduced in medical school (undergraduate and postgraduate) studies. Similarly, such stewardship plans should be introduced in other health professionals' curricula around the globe. The installation of antimicrobial stewardship in medical and other health professional courses raises the hope of prudent antimicrobial and overall prescribing practice among healthcare professionals. National and international drug regulatory authorities should introduce stringent antimicrobial prescribing and utilization policies. This will reduce morbidity and mortality not only among EF patients but also among other drug-resistant infectious diseases.
